# Removing outliers from the normative database improves regional atrophy detection in single-subject voxel-based morphometry

**DOI:** 10.1007/s00234-024-03304-3

**Published:** 2024-02-21

**Authors:** Vivian Schultz, Dennis M. Hedderich, Benita Schmitz-Koep, David Schinz, Claus Zimmer, Igor Yakushev, Ivayla Apostolova, Cansu Özden, Roland Opfer, Ralph Buchert

**Affiliations:** 1 Department of Neuroradiology, Klinikum Rechts Der Isar, Technical University of Munich, School of Medicine and Health, Ismaninger Str. 22, 81675 Munich, Germany; 2 Institute of Radiology, University Hospital Erlangen, Friedrich-Alexander-Universität Erlangen (FAU), Nürnberg, Germany; 3 Department of Nuclear Medicine, Klinikum Rechts Der Isar, Technical University of Munich, School of Medicine and Health, Munich, Germany; 4 Department of Diagnostic and Interventional Radiology and Nuclear Medicine, University Medical Center Hamburg-Eppendorf, Hamburg, Germany; 5 Jung Diagnostics GmbH, Hamburg, Germany

**Keywords:** Magnetic resonance imaging, Brain, Neurodegeneration, Voxel-based-morphometry, Normative database

## Abstract

**Purpose:**

Single-subject voxel-based morphometry (VBM) compares an individual T1-weighted MRI to a sample of normal MRI in a normative database (NDB) to detect regional atrophy. Outliers in the NDB might result in reduced sensitivity of VBM. The primary aim of the current study was to propose a method for outlier removal (“NDB cleaning”) and to test its impact on the performance of VBM for detection of Alzheimer’s disease (AD) and frontotemporal lobar degeneration (FTLD).

**Methods:**

T1-weighted MRI of 81 patients with biomarker-confirmed AD (*n* = 51) or FTLD (*n* = 30) and 37 healthy subjects with simultaneous FDG-PET/MRI were included as test dataset. Two different NDBs were used: a scanner-specific NDB (37 healthy controls from the test dataset) and a non-scanner-specific NDB comprising 164 normal T1-weighted MRI from 164 different MRI scanners. Three different quality metrics based on leave-one-out testing of the scans in the NDB were implemented. A scan was removed if it was an outlier with respect to one or more quality metrics. VBM maps generated with and without NDB cleaning were assessed visually for the presence of AD or FTLD.

**Results:**

Specificity of visual interpretation of the VBM maps for detection of AD or FTLD was 100% in all settings. Sensitivity was increased by NDB cleaning with both NDBs. The effect was statistically significant for the multiple-scanner NDB (from 0.47 [95%-CI 0.36–0.58] to 0.61 [0.49–0.71]).

**Conclusion:**

NDB cleaning has the potential to improve the sensitivity of VBM for the detection of AD or FTLD without increasing the risk of false positive findings.

**Supplementary Information:**

The online version contains supplementary material available at 10.1007/s00234-024-03304-3.

## Introduction

Made possible by methodological advances and drastically reduced processing times, automated brain volumetry from T1-weighted MRI in individual patients has recently entered clinical practice [1–3]. Many software tools provide z-scores of regional brain volumes relative to a normative database (NDB) of healthy individuals [4–6]. Some tools also use voxel-based morphometry (VBM) to generate voxel-wise z-score maps of gray matter (GM) density in individual subjects relative to the NDB. These voxel-wise VBM maps have been proven beneficial not only for the detection but also for the differentiation of neurodegenerative diseases [3, 7].

It is evident that the quality of the NDB can have considerable impact on the performance of single-subject VBM. Regarding the size of the NDB, for example, previous studies found that an NDB consisting of 20–30 scans can be used for single-subject VBM, but that an NDB with two to three times larger size might provide better sensitivity [8–10] and/or specificity [11].

Outliers in the NDB cause overestimation of the normal between-subjects variability (standard deviation) of GM density, which in turn causes underestimation of z-scores in single-subject VBM. As a result, true regional atrophy might fail to reach statistical significance according to a predefined cutoff on the regional z-scores. Against this background, the primary hypothesis in the current study was that removing outliers from the NDB (“NDB cleaning”) improves the sensitivity for the detection of Alzheimer’s disease (AD) or frontotemporal lobar degeneration (FTLD) by improving the power for the detection of regional atrophy. Among the dementing neurodegenerative diseases, suspected AD and suspected FTLD are by far the most common indications for VBM at most sites.

Furthermore, VBM is sensitive to the MRI scanner platform and to the details of the acquisition sequence [12–17]. Thus, an NDB of MRI scans acquired with the same MRI scanner and with exactly the same acquisition sequence as the individual MRI to be analyzed is the gold standard for single-subject VBM. However, a scanner-specific NDB (that has to be replaced after each relevant hardware and/or software update) is not available at many sites. The use of a scanner-specific NDB from another scanner (from another site) might cause VBM to detect scanner differences that might be difficult to discriminate from true atrophy in the VBM maps. This might be avoided by the use of a non-scanner-specific multiple-scanner NDB comprising normal scans from numerous different scanners and, thus, adequately representing the spectrum of scanners encountered in clinical practice. However, additional variability of no interest caused by between-scanner differences most likely reduces the sensitivity for the detection of true atrophy. Against this background, the secondary aim of the current study was to estimate the loss of VBM performance for detection of AD or FTLD with a multiple-scanner NDB compared to a scanner-specific NDB. This is clinically relevant, given that most commercially available software tools for MRI-based brain volumetry have implemented a multiple-scanner NDB [6].

## Materials and methods

### Test dataset

The test dataset for this retrospective study comprised 118 subjects, 81 patients (age 65.9 ± 8.2 years, 54% females) with AD (18 AD with amnestic dementia, 22 amnestic mild cognitive impairment (MCI) due to AD, 11 posterior cortical atrophy (PCA)) or FTLD (20 behavioral variant FTLD (bvFTLD), 10 semantic variant primary progressive aphasia (SD)) and 37 healthy controls (HC, 58.1 ± 10.9 years, 43% females). The subjects were included retrospectively from a previous prospective study on the relationship between local neuronal activity and the functional coupling among distributed brain regions [18] and from a previous retrospective study on the utility of single-subject VBM with a scanner- and sequence-specific NDB for the differential diagnosis of dementing neurodegenerative diseases in clinical practice [3]. The ground truth diagnoses had been established by dementia experts based on the results of biomarker information (FDG-PET, amyloid-PET, and/or CSF amyloid-β42, phosphorylated tau, and total tau), clinical examination, neuropsychological testing, and clinical follow-up.

In all subjects, simultaneous FDG-PET/MRI had been performed with the same PET-MRI hybrid system (Siemens Biograph mMR PET-MRI, Siemens Healthineers, Erlangen, Germany) using exactly the same acquisition sequence. Imaging included a 3D T1-weighted sequence with a resolution of 1 × 1 × 1 mm^3^ (TR = 2300 ms, TE = 2.98 ms, TI = 900 ms, flip angle = 9°).

### Normative databases

The scanner-specific single-scanner NDB (SSD) consisted of the 37 healthy controls from the test dataset.

The non-scanner-specific multiple-scanner NDB (MSD) comprised 3D T1-weighted MRI with 1 × 1 × 1 mm^3^ resolution from 164 subjects (64.1 ± 9.4 years, 57% females) acquired for unspecific symptoms (e.g., headache, dizziness) with 164 different MRI scanners at 164 different sites using acquisition sequences recommended by the scanner manufacturer. Imaging was performed at 3/1.5/1.0 Tesla in 47/114/3 cases (28.7/69.5/1.8%) using MRI scanners from three different manufacturers: Siemens (*n* = 110; Aera, Amira, Avanto, Espree, Galan, HarmonyExpert, MAGNETOM (Lumina, Vida, ESSENZA), Orian, Skyra (fit), Symphony (Tim), TrioTim, Verio), Philips (*n* = 40; Achieva (dStream), Ingenia, Intera, Panorama HFO), and GE (*n* = 14; DISCOVERY MR750, Optima MR450w, SIGNA (Hde, HDxt, Voyager)).

None of the patients had a history of or currently ongoing neurological or psychiatric disease. All scans were free of abnormalities beyond those expected for the patients’ age based on visual inspection by an experienced radiologist.

### Removal of outliers from the NDB

GM density maps in the anatomical space of the Montreal Neurological Institute (MNI) were obtained for each scan in the NDB as described in subsection “Single-subject voxel-based morphometry”. Then, a leave-one-out z-score map was computed for each GM map by voxel-wise application of the following formula:

1 $$\text{z}=\left(\mathrm{individual}\;\mathrm{GM}-\mathrm{mean}\;\mathrm{GM}\right)/\mathrm{standard}\;\mathrm{deviation}\;\mathrm{of}\;\mathrm{GM}$$

where mean and standard deviation of the GM density were computed over all GM density maps in the NDB excluding the individual GM map. The calculation of the z-score map was restricted to a standard GM mask predefined in MNI space (in order to avoid division by zero or very small numbers).

The following quality metrics were computed for each individual leave-one-out z-score map in a given NDB

2 $$\text{z}-\text{sum}=\mathrm{sum}\;\mathrm{of}\;\mathrm{all}\;z-\mathrm{scores}\left(\mathrm{absolute}\;\mathrm{value}\right)\mathrm{in}\;\mathrm{the}\;\mathrm{GM}\;\mathrm{mask}$$

3 $$\text{z}-\text{max}=\mathrm{maximum}\;\mathrm{of}\;\mathrm{all}\;z-\mathrm{scores}\;\left(\mathrm{absolute}\;\mathrm{value}\right)\;\mathrm{in}\;\mathrm{the}\;\mathrm{GM}\;\mathrm{mask}$$

4 $$\text{n}-\text{significant}=\mathrm{number}\;\mathrm{of}\;\mathrm{voxels}\;\mathrm{in}\;\mathrm{the}\;\mathrm{GM}\;\mathrm{mask}\;\mathrm{with}\;z\;\left(\mathrm{absolute}\;\mathrm{value}\right)>2.5$$

A scan in the NDB was considered an outlier with respect to one of these quality metrics if its corresponding value was equal to or larger than upper quartile + 1.0 * interquartile range of the quality metric in the NDB. A scan was considered an (overall) outlier if it was an outlier with respect to one or more of the quality metrics.

Identification and removal of outliers were performed separately for the two NDBs.

### Single-subject voxel-based morphometry (VBM)

Single-subject VBM relative to each of the four different NDBs (SSD and MSD before and after removal of outliers) was performed with the Biometrica analysis platform (jung diagnostics GmbH, Hamburg, Germany). In brief, the original 3D T1-weighted MRI was segmented into GM, white matter, and cerebrospinal fluid component images [15]. Spatial correspondence between the GM component image of the patient and the GM component images of the NDB was established via high dimensional non-linear image registration (DARTEL) [19]. The registered and modulated individual GM component image was smoothed by convolution with an isotropic Gaussian kernel of 8 mm full-width-at-half-maximum. After smoothing, a voxel-based two-sample *t* test of the individual smoothed GM component image against the smoothed GM component images of the NDB was carried out, resulting in a statistical t-map. Age and total intracranial volume (TIV) were taken into account as nuisance covariates. The TIV was estimated in each T1-weighted MRI by using a 3D convolutional neural network specifically trained for accurate and stable delineation of the TIV, in particular to avoid TIV overestimation occasionally observed with conventional methods [20, 21].

### Visual interpretation of individual VBM maps

**Fig. 1:**
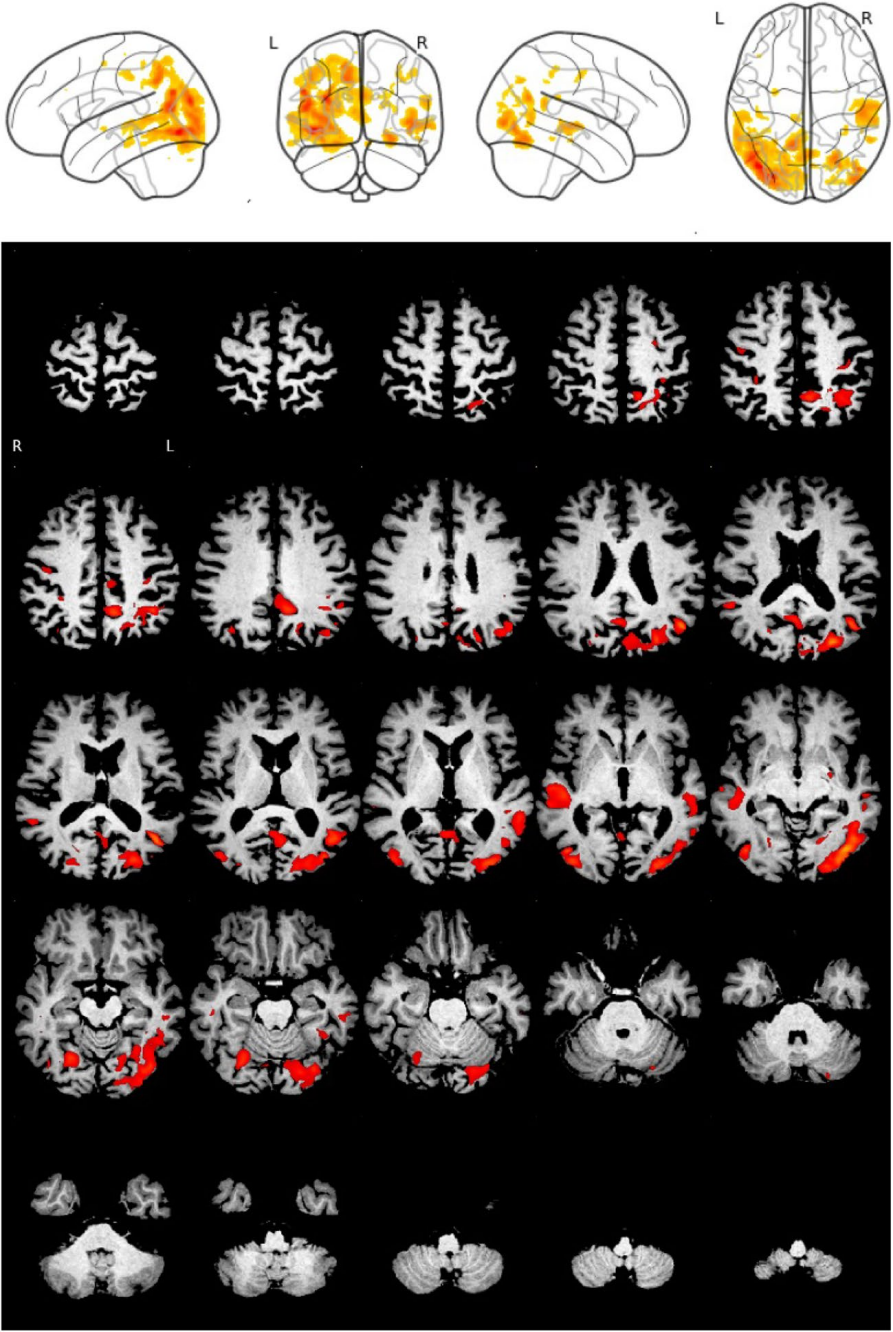
Standard display for visual interpretation of VBM maps. The example shows the VBM map of a 66-year-old patient with posterior cortical atrophy obtained with the full single-scanner normative database (SSD)

Individual VBM maps were thresholded at *p* = 0.005. For visual interpretation of the VBM maps, a standardized display was used that provided the thresholded VBM maps as color-coded overlay on axial slices and as a glass brain view in a one-page pdf document separately for each case (Fig. 1).

The VBM maps were interpreted by two neuroradiologists with 3 years and 8 years of experience in reading VBM maps of patients with suspected neurodegenerative disease. The readers were blinded for all clinical and biomarker information except age.

There were 472 different pdf documents (118 test cases × two NDBs × without or with removal of outliers). A copy was generated from each of these pdf documents to allow assessment of intra-reader variability of the visual interpretation. This resulted in 944 anonymized pdf documents that were presented in randomized order.

The readers were asked to use the following two-step approach for visual interpretation. First, the readers had to decide whether a neurodegenerative disorder was “present”, “absent”, or “uncertain”. If a neurodegenerative disorder was “present”, in the second step the reader categorized the atrophy pattern as AD or FTLD using criteria described previously [3] (Supplementary Fig. 1).

Cases with intra-reader discrepancy with respect to the detection of a neurodegenerative disease in the first step and/or categorization of the neurodegenerative disease in the second step were read a third time by the same reader to obtain an intra-reader consensus, separately for both readers. A joint reading session was used to resolve between-reader discrepancies of the intra-reader consensus to obtain a between-readers consensus.

### Statistical analysis

For each thresholded VBM map, the total volume of atrophy was computed by counting the number of voxels and then multiplying the total number of voxels by the volume of a single voxel. A general linear model for repeated measures was used to test the impact of NDB cleaning on the total volume of atrophy. NDB (SSD or MSD) and cleaning (without or with) were included as within-subject factors. The ground truth diagnosis (AD, FTLD, HC) was included in the model as between-subjects factor.

Cross tables and Cohen’s kappa were used to assess intra- and between-reader agreement of the visual interpretation and to assess the accuracy of the between-readers consensus relative to the clinical ground truth diagnosis, separately for each NDB. “Uncertain” cases were included in the “no neurodegenerative disease” category for statistical analyses to be as specific as possible.

IBM SPSS (version 27) was used for these statistical analyses. The threshold for statistical significance was set at two-sided *p* = 0.05.

Voxel-based group-level comparison of the GM density between the two NDBs, SSD and MSD, was performed with the heteroscedastic two-sample *t* test implemented in the statistical parametric mapping software package (version SPM12), separately before and after NDB cleaning. For rather sensitive detection of regional GM differences, the voxel-level significance threshold was set to one-sided *p* = 0.005 uncorrected for multiple comparisons. The minimum cluster size was fixed at 296 voxels (corresponding to 1-ml volume).

### Ethics statement

The retrospective use of the test dataset was approved by the ethics committee of the Technical University of Munich (Reference 176/18 s). The need for written informed consent was waived by the ethics committee due to the retrospective nature of the analysis.

The MRI data of the MSD had been transferred to jung-diagnostics GmbH under the terms and conditions of the European general data protection regulation for remote image analysis. Subsequently, the data had been anonymized. The need for written informed consent for the retrospective use of the anonymized data was waived by the ethics review board of the general medical council of the state of Hamburg, Germany.

## Results

There were seven outliers identified in the SSD (19%), 35 outliers in the MSD (21%) (Supplementary Fig. 2). In both NDBs, most of the outliers were an outlier with respect to the number of significant voxels (n-significant): six of seven (86%) overall outliers in the SSD and 25 of 35 (71%) overall outliers in the MSD. Outliers with respect to the two other quality metrics, z-sum and z-max, were less frequent: four and three of seven (57% and 43%) in the SSD, 15 and 15 of 35 (43%) in the MSD.

Demographical characteristics and TIV estimates in the two NDBs before and after removal of outliers are summarized in Table 1. Age differed significantly between the SSD and the MSD before (*p* < 0.001) but not after outlier removal. Sex and TIV did not differ significantly between the NDBs, neither before nor after removal of outliers.

**Table 1:** Demographics and total intracranial volume (TIV) in the two normative databases (NDBs) before and after removal of outliers

		Before outlier removal	After outlier removal
Single scanner database	*n*	37	30
	Mean age (SD)	58.12 (10.90) years	60.77 (10.22) years
	Sex	21 males, 16 females	14 males, 16 females
	Mean TIV (SD)	1381 (175) ml	1333 (153) ml
Multiple scanner database	*n*	164	129
	Mean age (SD)	64.07 (9.43) years	63.83 (9.68) years
	Sex	54 males, 71 females, 16 missing information	38 males, 67 females, 10 missing information
	Mean TIV (SD)	1372 (148) ml	1353 (125) ml

*SD* Standard deviation

**Fig. 2:**
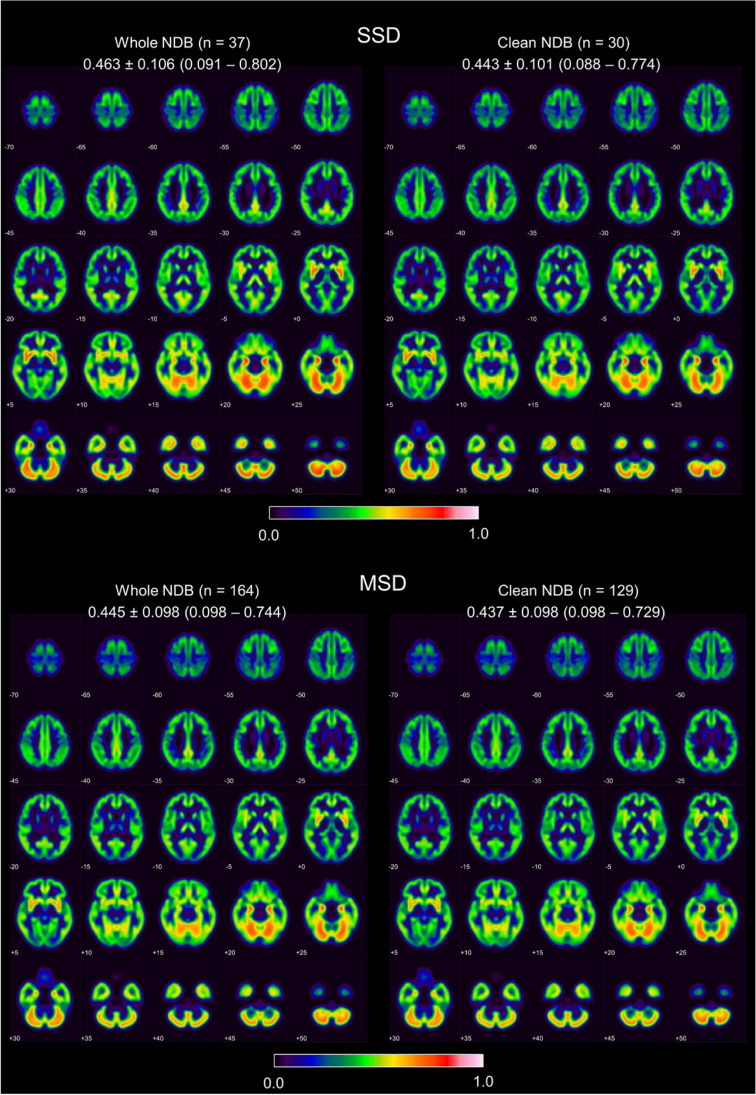
Voxel-wise mean of the GM density in the single-scanner normative database (SSD) (top) and in the multiple-scanner normative database (MSD) (bottom) before (left) and after (right) removal of outliers. Mean value ± standard deviation (range) is given for each setting

**Fig. 3:**
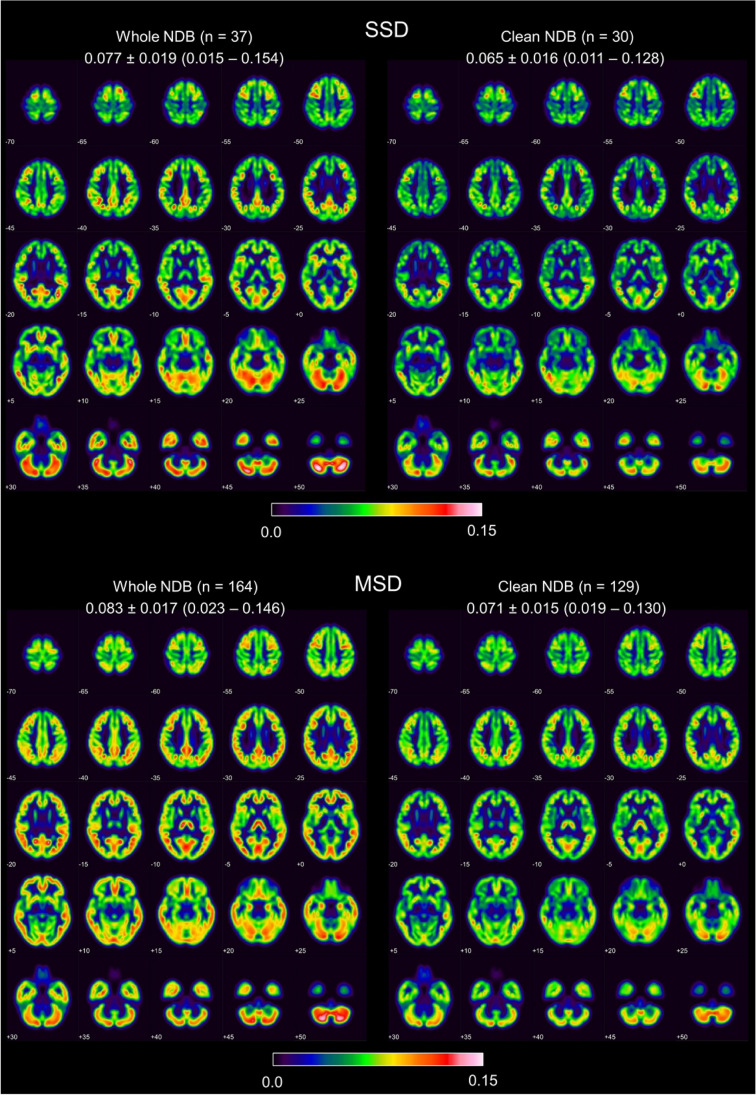
Voxel-wise standard deviation of the GM density in the single-scanner normative database (SSD) (top) and in the multiple-scanner normative database (MSD) (bottom) before (left) and after (right) removal of outliers. Mean value ± standard deviation (range) is given for each setting. The maximum of the color table was set to 0.15

Voxel-wise mean and voxel-wise standard deviation of the GM density in the two NDBs before and after removal of the outliers are shown in Figs. 2 and 3, respectively.

**Fig. 4:**
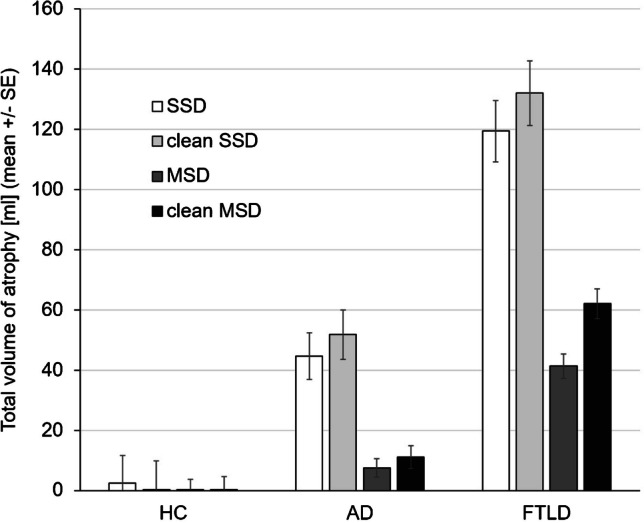
Mean value and standard error (SE) of the total volume of atrophy with the scanner-specific normative database (SSD) and with the multiple-scanner normative database (MSD) without and with removal of outliers (“cleaning”) in healthy controls (HC), patients with Alzheimer’s disease (AD), and patients with frontotemporal lobar degeneration (FTLD)

The general linear model for repeated measures revealed all within-subjects effects on the total volume of atrophy to be highly significant, including the interaction effects (cleaning: *p* < 0.0005, partial eta-squared *η*^2^ = 0.274; cleaning*ground truth: *p* < 0.0005, *η*^2^ = 0.268; cleaning*NDB*ground truth: *p* = 0.001, *η*^2^ = 0.122; NDB: *p* < 0.0005, *η*^2^ = 0.470; NDB*ground truth: *p* < 0.0005, *η*^2^ = 0.324). Thus, there was a significant effect of NDB cleaning that depended on the NDB, and the NDB dependence of the cleaning effect differed between HC, AD, and FTLD (Fig. 4). More precisely, the total volume of atrophy was larger after removal of outliers, more pronounced with the MSD, but only in patients with AD or FTLD, not in HC subjects.

In the first step of the eight visual reads of the VBM maps (two readers × two NDB × without or with cleaning), the number of cases that were categorized as “uncertain” with respect to the presence or absence of a neurodegenerative disease ranged between 4 and 14 (3–12%). After the uncertain cases were recategorized as “no neurodegenerative disease”, intra- and between-readers Cohen’s kappa of the binary visual interpretation of the VBM maps with respect to the presence of a neurodegenerative disease ranged between 0.868 and 1.0 and between 0.839 and 0.966, respectively (Supplementary Fig. 3). When the MSD was used, NDB cleaning resulted in reduction of intra- and between-readers agreement: intra-reader kappa from 0.972 ± 0.040 to 0.890 ± 0.030 (mean ± standard deviation across the two readers), between-readers kappa from 0.962 to 0.839 (Supplementary Fig. 3).

**Fig. 5:**
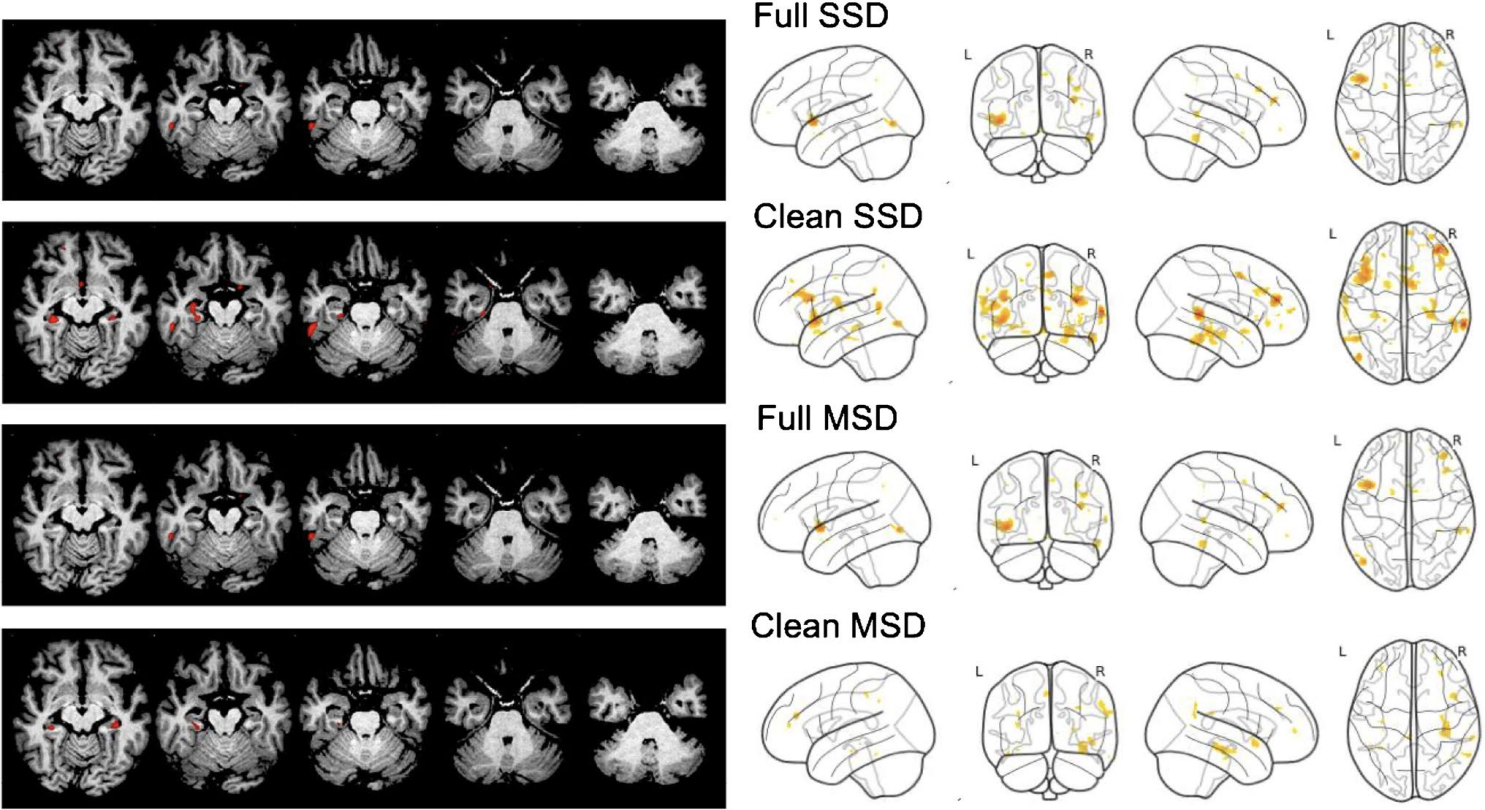
VBM maps of a patient with reference standard diagnosis of mild cognitive impairment (MCI) due to Alzheimer’s disease (AD) with (top-to-bottom) the scanner-specific normative database (SSD) before and after removal of outliers (“cleaning”) and with the multiple-scanner normative database (MSD) before and after removal of outliers. Removal of outliers led to a better delineation of hippocampal atrophy with the MSD whereas multiple (unspecific) atrophy clusters were detected with the MSD before removal of outliers as well as with the SSD independent of removal of outliers. The between-readers consensus of the visual interpretation was false negative (“no neurodegenerative disease”) with the SSD before and after removal of outliers and the MSD before removal of outliers and true positive (“neurodegenerative disease (AD)”) after removal of outliers with the MSD

Sensitivity, specificity, and predictive values of the consensus binary visual interpretation of the VBM maps for detection of any neurodegenerative disease (AD or FTLD) are given in Table 2. Specificity was 100% in all settings. Sensitivity was improved by NDB cleaning, particularly with the MSD (an exemplary case is given in Fig. 5). Performance estimates for the differentiation of AD from HC, FTLD from HC, and AD from FTLD are given in Table 3.

**Table 2:** Sensitivity, specificity, and predictive values for the detection of a neurodegenerative disease (Alzheimer’s disease (AD) or frontotemporal lobar degeneration (FTLD)) by visual interpretation of the single-subject VBM maps (consensus of the two readers) before and after removal of outliers from the normative database (NDB) (“cleaning”), separately for the single scanner NDB (SSD) and the multiple-scanner NDB (MSD)

NDB	Sensitivity [95% CI]	Specificity [95% CI]	PPV [95% CI]	NPV [95% CI]
SSD	0.82 [0.71–0.89]	1.0 [0.91–1.0]	1.0 [0.95–1.0]	0.71 [0.58–0.82]
Clean SSD	0.84 [0.74–0.91]	1.0 [0.91–1.0]	1.0 [0.95–1.0]	0.74 [0.60–0.84]
MSD	0.47 [0.36–0.58]	1.0 [0.91–1.0]	1.0 [0.91–1.0]	0.46 [0.36–0.57]
Clean MSD	0.61 [0.49–0.71]	1.0 [0.91–1.0]	1.0 [0.93–1.0]	0.54 [0.42–0.65]

*CI* Confidence interval, *PPV* Positive predictive value, *NPV* Negative predictive value

**Table 3:** Sensitivity, specificity, and predictive values for the discrimination of Alzheimer’s disease (AD) from healthy controls (HC), frontotemporal lobar degeneration (FTLD) from HC, and AD from FTLD by visual interpretation of the single-subject VBM maps (consensus of the two readers) before after removal of outliers from the normative database (NDB) (“cleaning”), separately for the single scanner NDB (SSD) and the multiple-scanner NDB (MSD)

	Sensitivity [95% CI]	Specificity [95% CI]	PPV [95% CI]	NPV [95% CI]
AD versus HC				
SSD	0.75 [0.61–0.85]	1.0 [0.91–1.0]	1.0 [0.90–1.0]	0.76 [0.62–0.85]
Clean SSD	0.77 [0.63–0.86]	1.0 [0.91–1.0]	1.0 [0.90–1.0]	0.77 [0.63–0.87]
MSD	0.27 [0.16–0.40]	1.0 [0.91–1.0]	1.0 [0.77–1.0]	0.51 [0.39–0.61]
Clean MSD	0.40 [0.27–0.54]	1.0 [0.91–1.0]	1.0 [0.83–1.0]	0.56 [0.44–0.67]
FTLD versus HC				
SSD	0.89 [0.72–0.96]	1.0 [0.91–1.0]	1.0 [0.86–1.0]	0.93 [0.80–0.97]
Clean SSD	0.92 [0.74–0.98]	1.0 [0.91–1.0]	1.0 [0.85–1.0]	0.95 [0.83–0.99]
MSD	0.77 [0.59–0.88]	1.0 [0.91–1.0]	1.0 [0.86–1.0]	0.84 [0.71–0.92]
Clean MSD	0.89 [0.73–0.96]	1.0 [0.91–1.0]	1.0 [0.87–1.0]	0.93 [0.80–0.97]
AD versus FTLD				
SSD	0.92 [0.80–0.97]	0.89 [0.72–0.96]	0.92 [0.80–0.97]	0.89 [0.72–0.96]
Clean SSD	0.86 [0.77–0.96]	0.79 [0.69–0.90]	0.86 [0.72–0.93]	0.85 [0.66–0.94]
MSD	0.86 [0.62–0.96]	1.0 [0.86–1.0]	1.0 [0.77–1.0]	0.92 [0.75–0.98]
Clean MSD	0.86 [0.67–0.95]	0.93 [0.77–0.98]	0.90 [0.71–0.97]	0.89 [0.73–0–96]

*AD* Alzheimer’s disease, *CI* Confidence interval, *FTLD* Frontotemporal lobar degeneration, *HC* Healthy controls, *NPV* Negative predictive value, *PPV* Positive predictive value

**Fig. 6:**
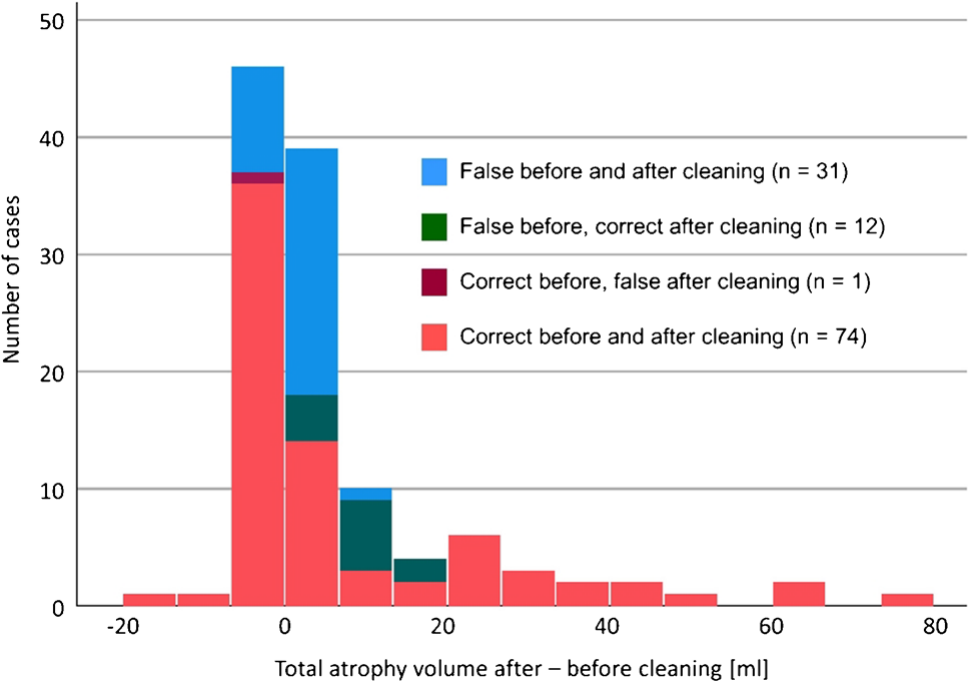
Histogram of the change in the total volume of atrophy by removal of outliers from the multiple-scanner normative database (MSD). The corresponding change in the consensus binary visual interpretation (presence versus absence of neurodegeneration) of the VBM maps is indicated by different colors

With the MSD, removal of outliers did not change the consensus binary visual interpretation in 105 of the 118 cases (89%). Among the 13 cases with discrepant consensus binary visual interpretation before and after removal of outliers, 12 (92%) were interpreted incorrectly when the full MSD was used but were interpreted correctly after removal of outliers from the MSD. It was vice versa in the remaining case (8%). The change from incorrect to correct interpretation in the 12 cases was driven by a 9 ± 5 mL increase of the total atrophy volume by NDB cleaning (Fig. 6). Total atrophy volume decreased by 4 ml by NDB cleaning in the case with change from correct to incorrect interpretation.

Statistical maps from the group-level comparison of the GM density between the two NDBs are shown in Supplementary Fig. 4. Prior to NDB cleaning, there were several clusters with significantly higher GM density in the SSD compared to the MSD, comprising a total volume of 128.0 ml. There were no clusters with significantly lower GM density in the SSD. After NDB cleaning, the number and the size of clusters with significantly higher GM density in the SSD decreased (from 128.0-ml to 35.8-ml total volume). In addition, a small (5.6 ml) cluster of significantly lower GM density in the SSD occurred after NDB cleaning.

## Discussion

The primary finding of this study was an improvement of sensitivity for the detection of AD or FTLD without increased risk of false positive findings in single-subject VBM by removing outliers from the NDB, in line with the primary hypothesis.

The benefit from removal of outliers was more pronounced for the MSD than for the SSD. This might be related to the difference in sample size of these NDBs. Whereas the SSD was relatively small (*n* = 37), the MSD was rather large (*n* = 164). This suggests that loss of statistical power associated with further reduction in size of small NDBs by removal of outliers might offset the benefit from avoiding overestimation of the normal variability (standard deviation) of regional gray matter density by the outliers [10].

The effect of NDB cleaning by removing outliers in general differs between different NDBs. This is not a limitation of the proposed NDB cleaning method but a necessary feature. First, the proportion of outliers identified by the proposed NDB cleaning method should depend on the homogeneity of the NDB. In particular, if the scans have been selected carefully in order to avoid outliers right from the start, NDB cleaning should remove fewer scans than when the original NDB has been put together less carefully. Second, the impact of the NDB cleaning on the power of VBM to detect regional atrophy should depend not only on the number of outliers that have been removed but also on their severity. This also might have contributed to the fact that the sensitivity improvement by NDB cleaning was more pronounced with the MSD than with the SSD (due to more severe outliers in the MSD than in the SSD; Supplementary Fig. 2). The potential benefit from removing “mild” outliers from a small NDB might be offset by the loss of statistical power due to the reduced sample size of the cleaned NDB. In contrast, removing a few very severe outliers might be beneficial also in case of small NDBs.

A scan in the NDB was considered an outlier if its corresponding value of at least one of three quality metrics was equal to or larger than upper quartile + 1.0 * interquartile range of the quality metric in the NDB. The rationale for using a non-parametric rule based on quartiles rather than a parametric rule based on standard deviations was to reduce the sensitivity of the outlier identification to the exact GM distributions in the NDB. The specific cutoff selected for the current study is rather sensitive, which resulted in the removal of about 20% of the scans from both NDBs. If the NDB is rather small from the beginning, a more restrictive outlier definition might be applied (e.g., upper quartile + 1.5 * interquartile range as cutoff and/or outlier with respect to more than one quality metric). It might be worth noting that estimates of the normal standard deviation are particularly sensitive to outliers in the NDB, more sensitive than estimates of the normal mean. This is due to the fact that estimation of the standard deviation is based on the *squared* differences from the mean. Thus, even removal of a small percentage of (strong) outliers can have a relevant impact on the estimates of the standard deviation. Applying non-parametric permutation methods instead of the parametric t-statistics can also reduce the sensitivity of voxel-based testing to outliers, but they are rarely used for VBM [11, 22, 23].

Visual inspection of the leave-one-out z-score maps from individual outliers suggested that outliers caused by a specific MR scanner and/or an unusual acquisition sequence are mainly identified by the z-sum metric (Supplementary Fig. 5). Outliers associated with characteristics of individual subjects are mainly identified by the z-max and by the n-significant metric, where z-max is more sensitive to focal effects and n-significant is more sensitive to less severe but spatially more extended differences. Thus, the three quality metrics are rather complementary.

Regarding the question whether or not to exclude cases from the NDB that were “mathematically” identified as outliers based on the proposed quality metrics, we believe that outliers in an MSD caused by the use of a specific MR system and/or an unusual acquisition sequence in general should be excluded in order to improve the sensitivity of MSD-VBM (Supplementary Fig. 6). This is less clear for outliers due to unusual focal or lobar GM density in individual subjects, provided these are actually normal physiological variants in healthy subjects (rather than being caused by an unrecognized disease). Excluding normal physiological variants from the NDB also increases the sensitivity of VBM to detect AD and FTLD. However, it will increase the sensitivity for the detection of the normal physiological variants, too. Whether this is desirable depends on the setting. Furthermore, particularly young or particularly old age of a subject might also result in identification of the scan as an outlier, as GM density changes with healthy aging. However, it might be desirable to include these scans in the NDB in order to cover a large age range.

The accuracy of single-subject VBM for the discrimination between AD and FTLD was lower after NDB cleaning (Table 3). This might appear surprising at first glance, but it is a direct consequence of the increased sensitivity for the detection of regional atrophy by NDB cleaning that resulted in some rather small clusters of atrophy detected by VBM after NDB cleaning but not before. Furthermore, differentiation of AD and FTLD was complicated in this study by the fact that the test dataset included different subtypes of both, AD and FTLD. In particular, mild atrophy in the anterior temporal lobe including hippocampus, amygdala, temporal pole, and lateral parts of the anterior temporal lobe (inferior and middle temporal gyrus) in the SD variant of FTLD can be difficult to discriminate from isolated mild mesiotemporal atrophy in AD (in the MCI stage) [3, 24]. In fact, two of the SD cases with rather small atrophy volumes were misclassified as AD based on the SSD-VBM map after removal of outliers. Furthermore, it is not possible to differentiate between mesio- and lateral temporal clusters in the lateral glass brain views (Fig. 1), which may have contributed to an increased uncertainty in differentiating AD and SD. This uncertainty might be avoided by including medial render views in the standardized display (to be tested in future studies).

Unexpectedly, intra- and between-readers agreement were slightly lower after NDB cleaning, although still excellent (kappa > 0.80). This effect was most pronounced with the MSD (Supplementary Fig. 3). It was caused by rather small atrophy clusters detected with the MSD after removal of outliers in cases with blank VBM map with the full MSD. These small atrophy clusters contributed to improved sensitivity for the detection of a neurodegenerative disease, but they also caused reduction of intra- and between-readers agreement (in case of an empty VBM map there is no alternative to categorizing it as “no neurodegenerative disease”).

Regarding the secondary aim of the current study, the multiple-scanner NDB was clearly outperformed by the scanner-specific NDB in terms of diagnostic accuracy of VBM, as expected. Although harmonization of acquisition sequences across different MRI scanners is a key current research focus, a scanner-specific NDB is still the gold standard for VBM. However, if a scanner-specific NDB is not available, a multiple-scanner NDB comprising scans of healthy controls from a large set of different scanners might be preferred over a scanner-specific NDB from another scanner in order to avoid misinterpretation of scanner-differences (as atrophy) detected by VBM. This was confirmed by the current study, as the MSD did not cause any false positive cases, in line with the fact that group-level GM differences between the MSD and the SSD were restricted to rather small brain regions, even at the sensitive uncorrected *p* = 0.005 significance threshold, particularly after NDB cleaning (Supplementary Fig. 4). Furthermore, the current study demonstrated that clinically useful sensitivity of VBM can be achieved with a multiple-scanner NDB as well. These findings support the use of a cleaned MSD for VBM analyses when an SSD is not available.

A secondary finding of this study was higher sensitivity of VBM for the detection of FTLD compared to AD. Possible explanations include that the FTLD patients were in more advanced stages of neurodegeneration compared to the AD patients (in line with the large proportion of patients with MCI among the AD patients 22 of 51, 43%), despite the fact that overall cognitive performance as measured by the MMSE did not differ between both groups. This was corroborated by the markedly larger total volume of atrophy in the FTLD patients (Fig. 4).

The novel NDB cleaning method is not restricted to VBM. It is easily adaptable to voxel-based statistical testing of other brain imaging modalities including FDG-, amyloid-, and tau-PET. Finally, the novel database cleaning method might be extended to the cleaning of training and validation datasets for deep learning-based approaches.

Limitations of the current study include the following. First, the healthy control scans in the test dataset were identical to the healthy control scans in the SSD, which might have caused some bias in favor of the SSD for VBM. Thus, the loss of sensitivity of VBM by the use of a multiple-scanner NDB relative to a scanner-specific NDB might have been overestimated in this study. However, the primary aim of this study, to investigate the effect of removal of outliers on diagnostic accuracy as a method per se is not limited through this fact. Second, there was a statistically significant age difference between the SSD and the MSD before removal of outliers, and it is well known that GM volumes depend on age [25], and the age dependency varies between brain regions [26]. However, the mean age difference was rather small (6 years), and age was taken into account as nuisance covariate in all single-subject VBM analyses. Thus, the age difference between the two NDBs most likely did not have a major impact on the current findings. Furthermore, while limitations of the age matching of the two NDBs might have affected their comparison regarding the VBM-based detection of regional atrophy, it most likely had no relevant impact on the primary finding of the study, namely increased sensitivity of VBM for detection of regional atrophy by NDB cleaning. Third, the test dataset included rather highly selected subjects and, therefore, might not be representative of clinical practice. In particular, the majority of the healthy control subjects had been recruited from the community for a prospective FDG-PET/fMRI study [18]. This might have contributed to the lack of false positive findings in the single-subject VBM analyses (in addition to the inclusion of “uncertain” cases in the “no neurodegenerative disease” category). Finally, the patients in the test dataset had AD or FTLD. Thus, the current findings regarding VBM performance apply to AD and FTLD only. However, the improvement in sensitivity by removal of outliers was similar for AD and FTLD, suggesting that this finding can be transferred to other diseases as the proposed method does not make any assumptions regarding the atrophy patterns to be detected. However, this needs to be tested in further studies. The maps of the voxel-wise standard deviation of GM density in the NDBs of this study demonstrate regional differences in the normal variability that might result in regional differences in the power of VBM to detect local atrophy (Fig. 3).

In conclusion, systematic removal of outliers from the NDB used as reference for voxel-based statistical testing has the potential to increase the sensitivity of single-subject VBM for the detection of AD or FTLD by increased sensitivity for the detection of regional atrophy. Furthermore, if a scanner-specific NDB is not available, a non-scanner-specific multiple-scanner NDB allows unbiased single-subject VBM without increased risk of false positive findings but at the expense of reduced sensitivity.

### Supplementary Information

Below is the link to the electronic supplementary material.

Supplementary file1 (DOCX 1844 KB)

## Data Availability

The data that support the findings of this study are available upon reasonable request.

## Abbreviations

AD: Alzheimer’s disease
bvFTLD: Behavioral variant frontotemporal lobar degeneration
CSF: Cerebrospinal fluid
FDG-PET: Fluorine-18-fluorodeoxyglucose positron emission tomography
FTLD: Frontotemporal lobar degeneration
GM: Gray matter
HC: Healthy controls
MCI: Mild cognitive impairment
MMSE: Mini Mental State Examination
MNI: Montreal Neurological Institute
MRI: Magnetic resonance imaging
MSD: Multiple-scanner normative database
NDB: Normative database
PCA: Posterior cortical atrophy
SD: Semantic variant primary progressive aphasia
SPSS: Statistical Package for Social Sciences
SSD: Single-scanner normative database
TE: Echo time
TI: Inversion time
TIV: Total intracranial volume
TR: Repetition time
VBM: Voxel-based morphometry
