# Supplementary material for: Removing outliers from the normative database improves regional atrophy detection in single-subject voxel-based morphometry
Source: Neuroradiology. 2024 Feb 21;66(4):507–19. doi: 10.1007/s00234-024-03304-3 (PMC10937771; doi:10.1007/s00234-024-03304-3)
Supplement: Supplementary file 1 — Supplementary file1 (DOCX 1844 KB) [file 234_2024_3304_MOESM1_ESM.docx]

**Supplementary material**

**Removing outliers from the normative database improves regional atrophy detection in single-subject voxel-based morphometry**

**Neuroradiology**

**
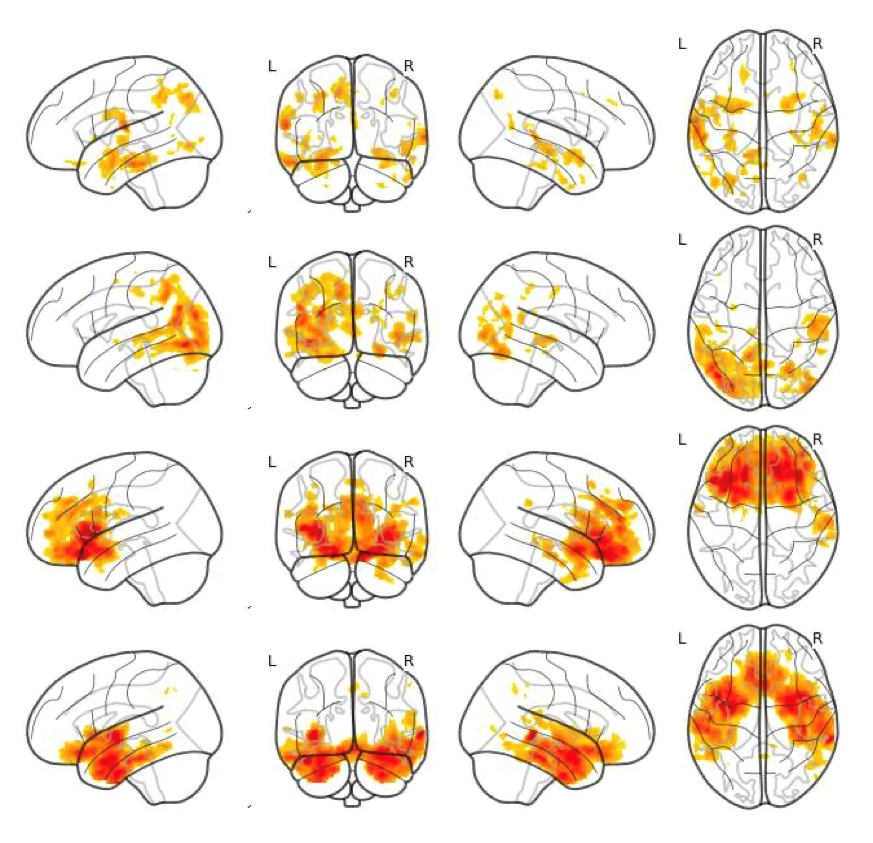
**

**Supplementary Fig. 1** 3D renderings of age- and TIV-corrected GM loss at uncorrected p < 0.005 typical for (top-to-bottom): (i) amnestic Alzheimer’s disease: atrophy with a symmetric or asymmetric pattern of the medial temporal lobe, posterior cingulate cortex, and temporoparietal junction; frontal atrophy is possible [1], (ii) posterior cortical atrophy: atrophy with a symmetric or asymmetric pattern of the posterior temporal lobe and the parietal lobe with predominant involvement of the occipital lobe [2], (iii) behavioral variant frontotemporal lobar degeneration: atrophy with a symmetric or asymmetric pattern of the frontal and anterior temporal lobes, with predominant involvement of the frontal lobes or similar atrophy of frontal and anterior temporal lobes [3], and (iv) semantic variant primary progressive aphasia: atrophy with a symmetric or asymmetric pattern of the frontal and anterior temporal lobes, with predominant involvement of the anterior temporal lobe, often most pronounced in the left hemisphere [4]

**
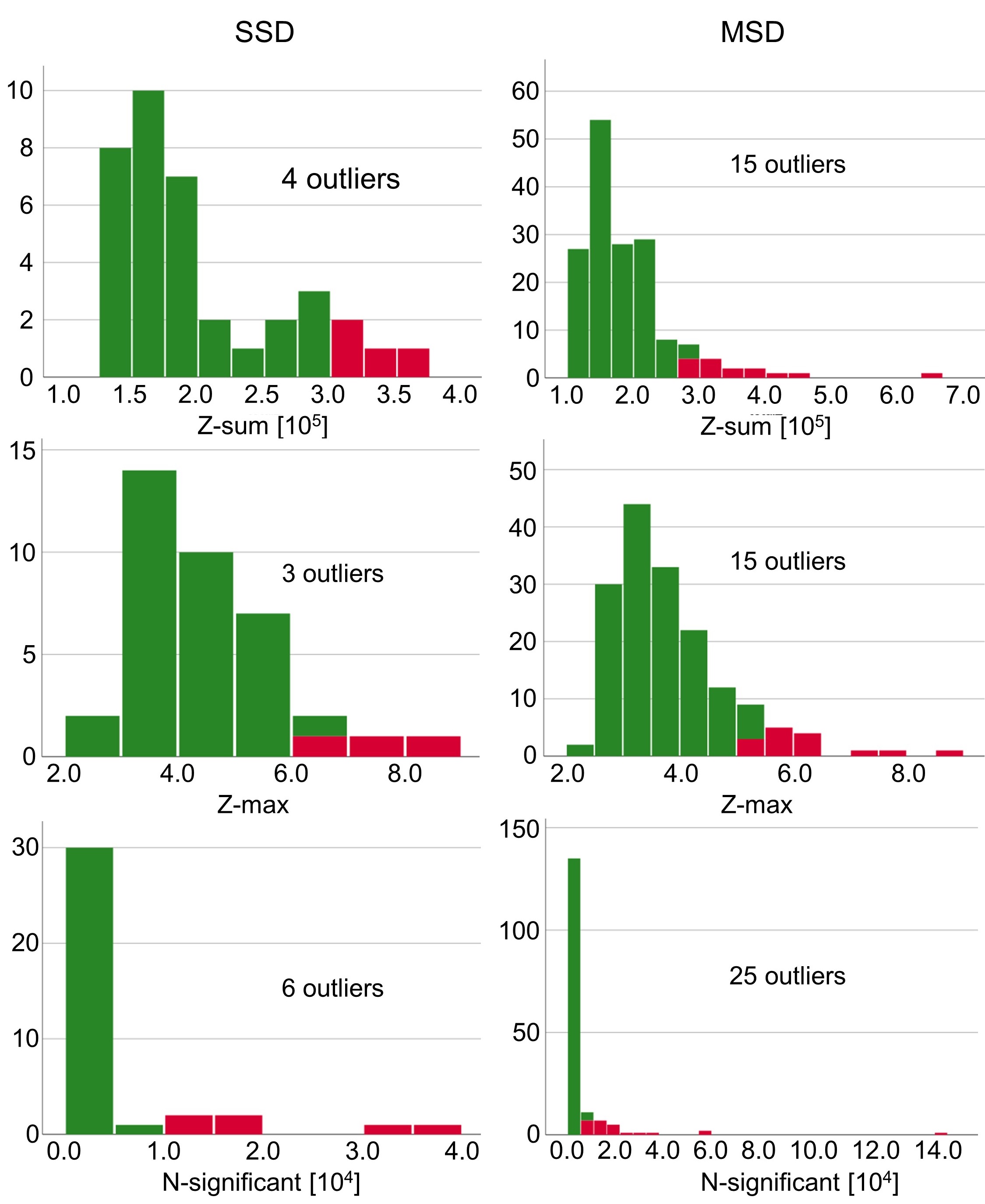
**

**Supplementary Fig. 2** Histograms of the three quality metrics used to identify outliers (red) in the scanner-specific normative database (SSD, left) and in the multiple-scanner normative database (MSD, right)

**
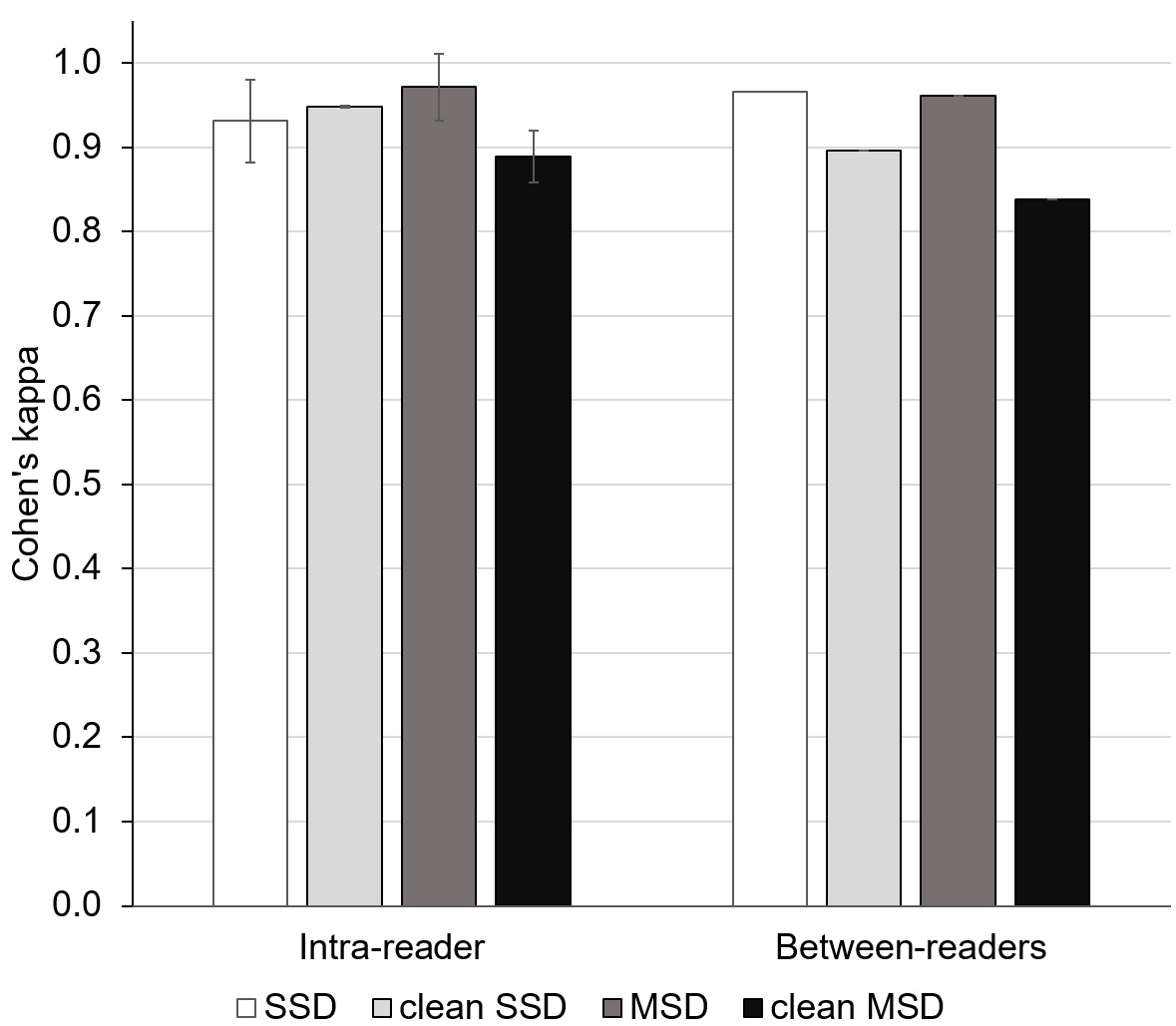
**

**Supplementary Fig. 3** Cohen’s kappa of intra- and between-readers agreement of the binary visual interpretation of the VBM maps with respect to the presence of a neurodegenerative disease (Alzheimer’s disease or frontotemporal lobar degeneration). Mean value and standard deviation of Cohen’s kappa of intra-reader agreement was computed across the two readers

*
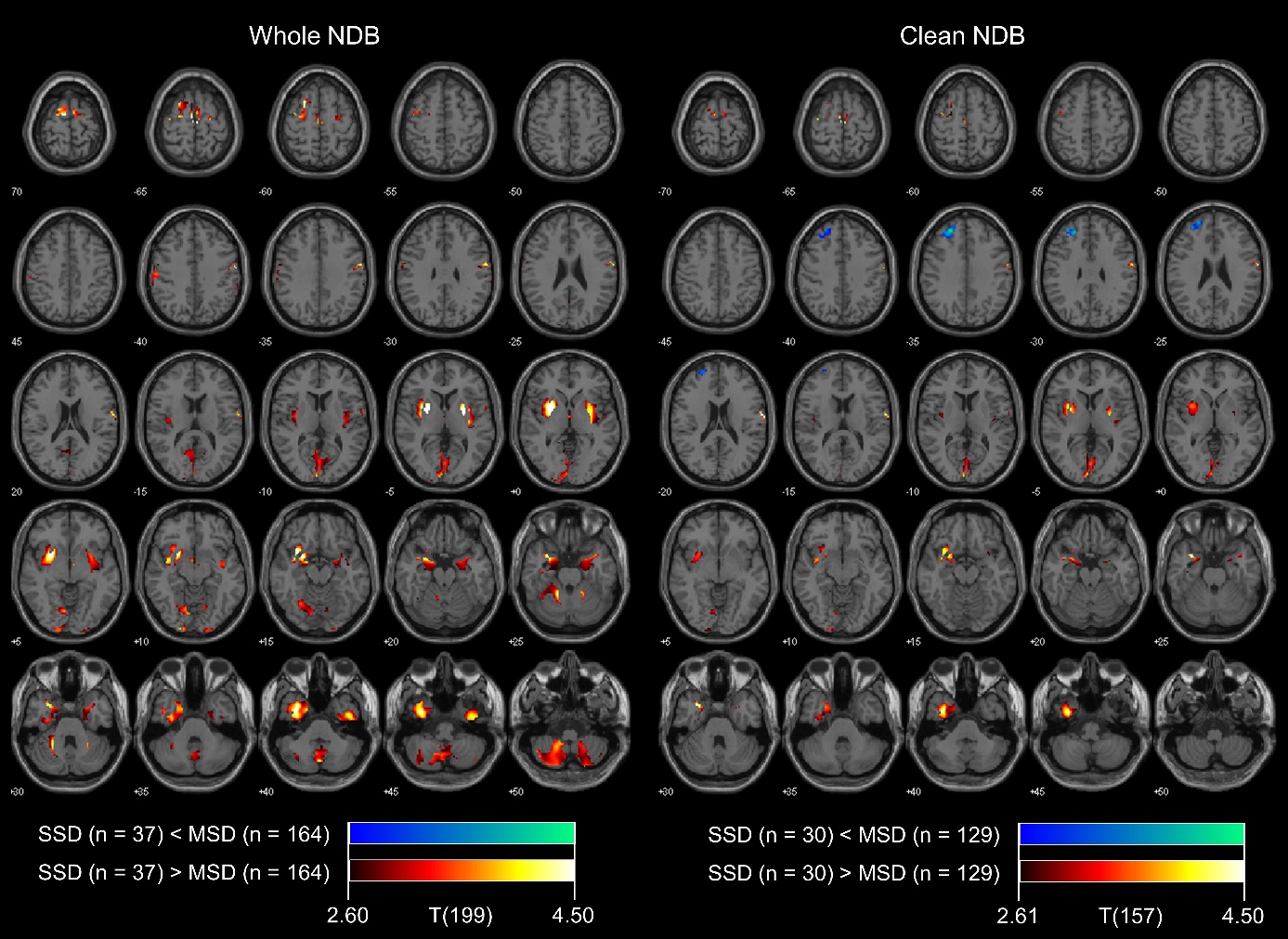
*

**Supplementary Fig. 4** Statistical parametric maps of the voxel-wise difference of the mean GM density between the SSD and the MSD before (left) and after (right) removal of outliers (“database cleaning”). For rather sensitive detection of differences between the two NDBs, the voxel level significance threshold was set to p = 0.005 uncorrected for multiple comparisons. The minimum cluster size was fixed at 296 voxels (corresponding to 1 ml volume). Voxels with significantly higher GM density in the SSD compared to the MSD are displayed with the hot color table, voxels with significantly lower GM density in the SSD are displayed with the winter color table. The statistical maps are overlaid to the single subject template of SPM12

**
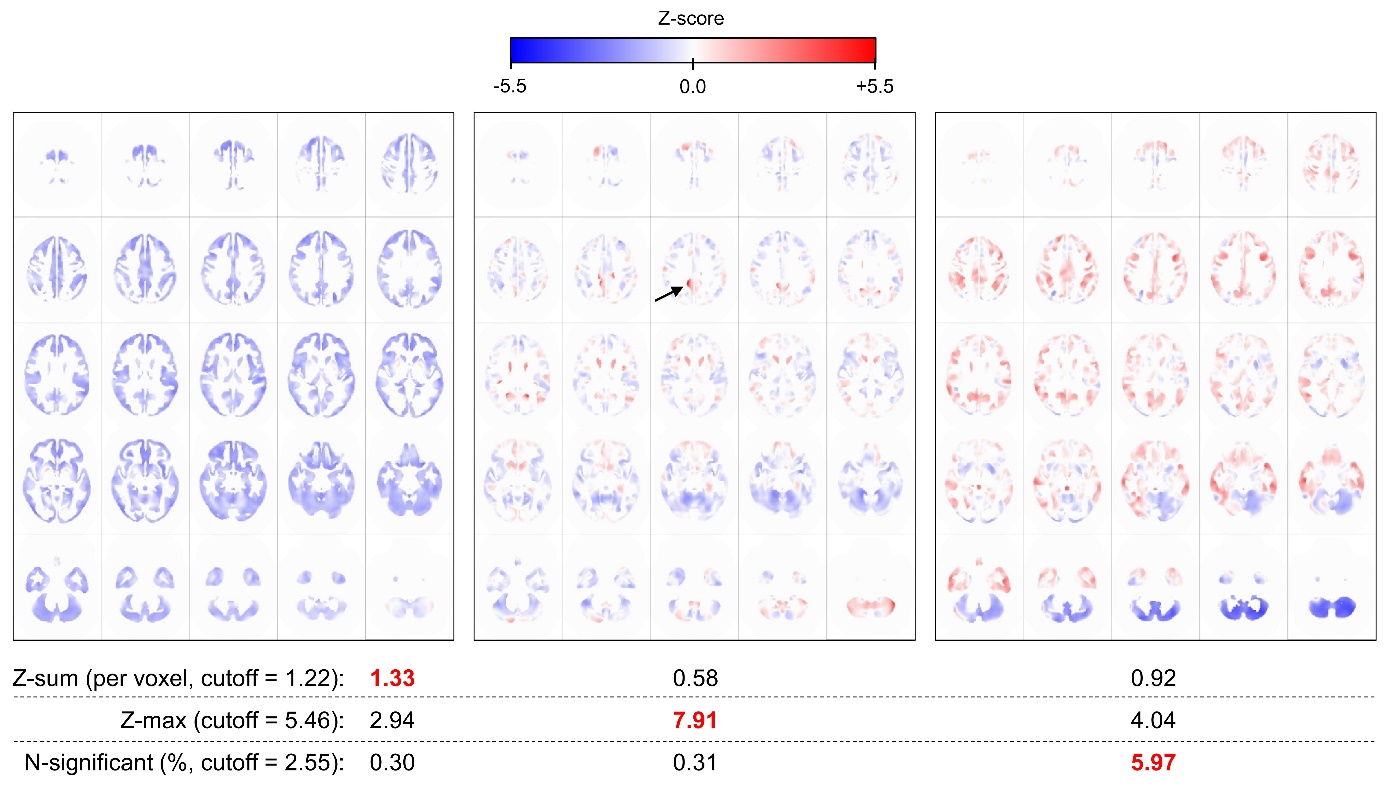
**

**Supplementary Fig. 5** Z-score maps for 3 different scans identified as outlier by leave-one-out testing of the GM density in the MSD. The left scan was an outlier with respect to the sum of all z-scores (absolute value) in the GM mask (z-sum): the z-sum per voxel in the GM mask was 1.33, that is, larger than the cutoff of 1.22 z-score points per voxel set at the upper quartile + 1.0 * interquartile range of the z-sum across all 164 scans in the MSD. The large z-sum was caused by rather uniformly reduced GM density throughout the whole brain. This suggests that the outlier was associated with the specific MR scanner and/or an unusual acquisition sequence used for this 3D T1-weighted MRI rather than being associated with the individual subject. The middle scan was an outlier with respect to the maximum of all z-scores (absolute value) in the GM mask (z-max): z-max of 7.91 was reached in a focal z-score hot spot in the posterior cingulate cortex (black arrow), clearly above the cutoff of 2.94. Otherwise, the z-score map switched between regions with positive z-scores and regions with negative z-scores, both with low amplitude. This suggests that the outlier was caused by pronounced focal deviation of the GM density in the posterior cingulate cortex in the individual subject rather than by the MR scanner or by an unusual acquisition sequence. The right scan was an outlier with respect to the number of voxels in the GM mask with z (absolute value) > 2.5 (n-significant): 5.97% of all voxels in the GM mask showed a significantly different GM density, considerably above the cutoff at 0.30%. This was mainly driven by low z-scores in the whole bilateral cerebellum, suggesting that the outlier was mainly caused by particularly low cerebellar GM density in the individual subject

*
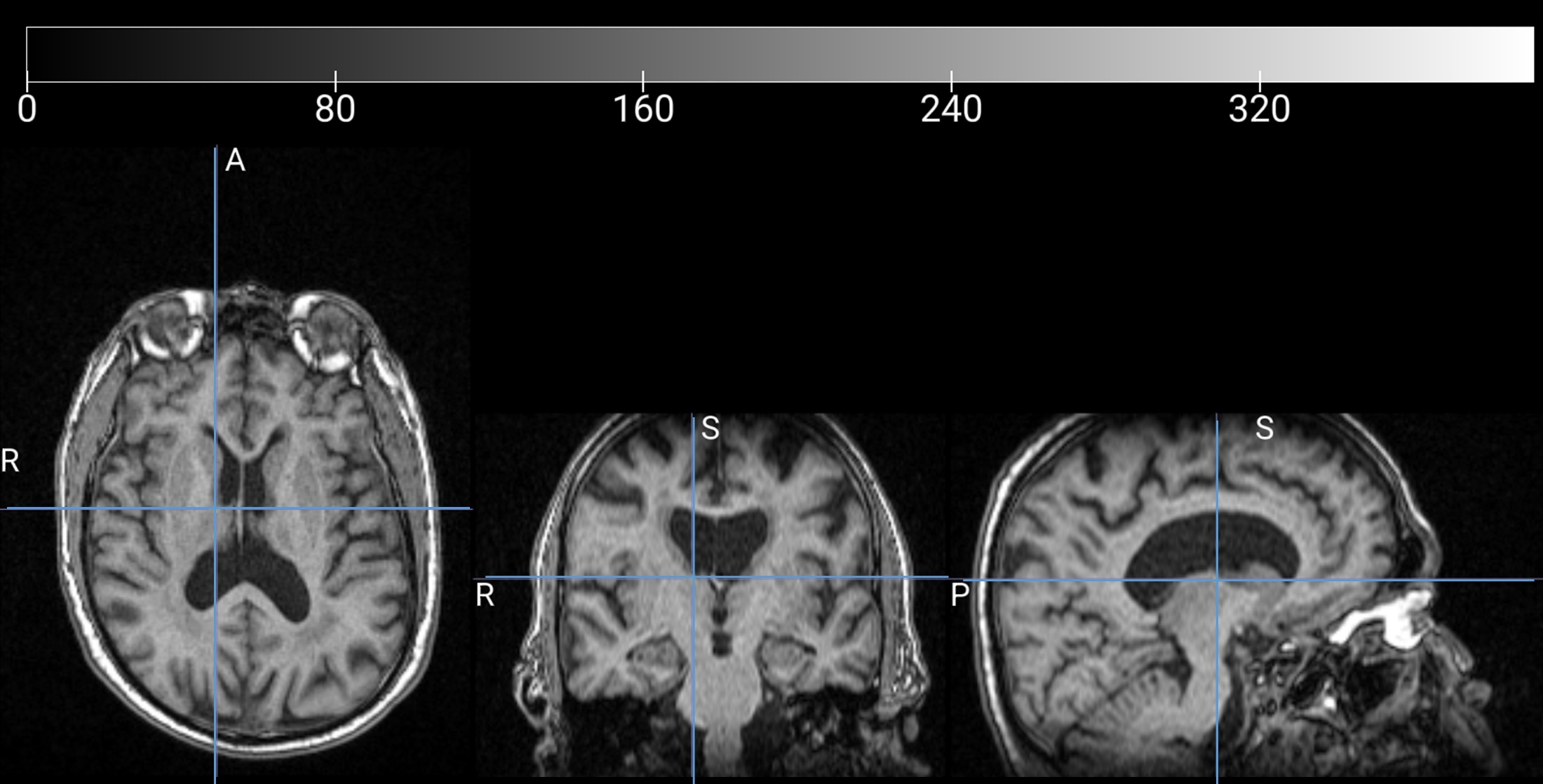
*

**Supplementary Fig. 6** Axial, coronal and sagittal images from a scan identified as an outlier in the multiple-scanner normative database. The scan is limited by a too narrow field-of-view, a rather high noise level and a somewhat reduced gray-to-white matter contrast.

**References to the Supplementary Material**

1. Whitwell JL, Jack CR, Przybelski SA et al (2011) Temporoparietal atrophy: a marker of AD pathology independent of clinical diagnosis. Neurobiol Aging 32:1531-1541. <https://doi.org/10.1016/j.neurobiolaging.2009.10.012>

2. Lehmann M, Crutch SJ, Ridgway GR et al (2011) Cortical thickness and voxel-based morphometry in posterior cortical atrophy and typical Alzheimer's disease. Neurobiol Aging 32:1466-1476. <https://doi.org/10.1016/j.neurobiolaging.2009.08.017>

3. Rohrer JD (2012) Structural brain imaging in frontotemporal dementia. Biochim Biophys Acta 1822:325-332. <https://doi.org/10.1016/j.bbadis.2011.07.014>

4. Rohrer JD, Warren JD, Modat M et al (2009) Patterns of cortical thinning in the language variants of frontotemporal lobar degeneration. Neurology 72:1562-1569. <https://doi.org/10.1212/WNL.0b013e3181a4124e>
